# Effect of Three Types of Activities on Improving Mood and Enjoyment in a Brief Online Depression Study

**DOI:** 10.1155/2020/1387832

**Published:** 2020-01-25

**Authors:** Caitriona Tilden, Melissa H. Bond, Taylor N. Stephens, Tyler Lyckberg, Ricardo F. Muñoz, Eduardo L. Bunge

**Affiliations:** ^1^Palo Alto University, CA, USA; ^2^Children and Adolescents Psychotherapy and Technology (CAPT) Lab, CA, USA; ^3^Institute for International Internet Interventions for Health (i4Health), CA, USA

## Abstract

The goal of this study is to determine whether different types of activities have a differential effect on mood and enjoyment. *Methods.* A secondary analysis of the data of 754 participants (*M*_age_ = 35.8 years, *SD*_age_ = 12.6; *M*_PHQ-9_ = 7.6, *SD*_PHQ-9_ = 7.0) who were recruited via Amazon Mechanical Turk (AMT) to participate in a brief online study. Participants completed an activity log and reported retrospectively about three types of activities (Pleasant, Meaningful, and Mastery) at baseline and one week follow-up. A mixed effects ANOVA was used to analyze the effect of weekly activities on mood, and a temporal analysis model was used to test for the effect of daily activities on enjoyment. *Results.* Participants who reported higher number of Mastery activities for the week had higher mood ratings at follow-up (F (1, 39) = 4.89, *p* < .05), regardless of depression status at baseline. Pleasant and Meaningful activities did not have a significant effect on mood. Daily engagement in any of the three activity types increased enjoyment of that day (Pleasant: *b* = 0.312, *t *(1811) = 46.73, *p* < .001; Meaningful: *b* = 0.254, *t *(1814) = 11.65, *p* < .001; Mastery: *b* = 0.290, *t *(1816) = 13.07, *p* < .001]. *Conclusions.* These findings contribute to the understanding on how brief behavioral activation interventions delivered online may influence participants' mood and enjoyment, and can inform clinicians' recommendations about types of activities.

## 1. Introduction

Depression affected approximately 4.4% of the global population, or 322 million people, in 2015 [1]. In the Global Burden of Disease (GBD) 2010 study, depressive disorders were found to be one of the leading causes of years lived with disability and disability adjusted life years, where Major Depressive Disorder (MDD) was the main contributor [2]. MDD ranked in the top ten leading causes of years lived with disability for more than 190 countries and territories in 2016 [3].

Behavioral activation (BA) is an intervention aimed at supporting individuals in engaging in activities which will increase the positive reinforcement an individual gets from their environment. BA has been shown to have strong empirical support in the treatment of depression [4]. However, the unique role of different types of activities (Pleasant, Meaningful, and Mastery activities) on individuals' mood has not been established. The behavioral theory of depression states that the onset of depression is associated with a decrease in pleasant events or an increase in aversive events [5, 6]. BA has been defined as an intervention aimed at increasing the amount of positive reinforcement available in a person's environment while decreasing activities that maintain depression. Lewinsohn and colleagues [7] developed BA as a stand-alone therapy that seeks to help people re-engage in activities. Engaging people in activities helps to combat depression by offering a source of positive reinforcement that serves as a natural antidepressant.

Life-enhancing activities can be grouped into at least three categories: Pleasant, Mastery, and Meaningful. Pleasant activities refer to activities which promote feelings of enjoyment, amusement, or pleasure derived from any given activity; sometimes the eliciting of even a mild satisfaction has been shown to restore morale and produce optimism [8]. Discovering sources of pleasurable activities is a first step in facilitating a patient's shift from a depressive state to a healthy mood state, although Pleasant activities alone may not be successful in producing a lasting change in depressive states.

Mastery activities are those that utilize strategies to incorporate feedback and corrective procedures into learning where conditions of success are outlined before instruction begins [9]. Mastery refers to a sense of competence and accomplishment when completing a task [8] and includes breaking larger overwhelming tasks into more manageable units.

Meaningful activities are defined as those which offer a state of optimal experience, marked by reported feelings of living up to one's values, which makes the experience genuinely satisfying. Happiness does not depend on outside events alone, but rather on how we interpret and respond to them [10].

Research has identified gender and age differences in the symptomatology profiles of depression [11]. There are differences in the way that men and women are likely to experience depression and the ways in which a BA intervention may impact their experience [12]. Age also plays a crucial role in BA for depression, wherein activities that address age-appropriate psychosocial needs related to the quality of an experience are significantly more Meaningful [13].

The aim of this study was to analyze the effect of frequency and the type of activities on enjoyment and mood. More specifically to determine whether different types of activities (Pleasant, Meaningful, and Mastery activities) have a differential effect on mood and enjoyment levels.

## 2. Methods

### 2.1. Participants

Participants were recruited through Amazon Mechanical Turk (AMT) to participate in an online brief Behavioral Activation intervention for mood management study previously published [14]. The current study is a secondary analysis of these data. Amazon Mechanical Turk (AMT) is an online platform that can be used to recruit participants rapidly for a minimal cost [15, 16]. AMT workers receive small payments per survey and AMT data gathered for clinical studies have shown to be high quality [17] for a full discussion see Buhrmester et al. [15]. A total of 754 participants consented, 70 of these were lost to attrition. The final sample size was 684 (*M*_age_ = 35.8 years old, *SD*_age_ = 12.6, Range_age_ 18–77, 67.5% female, *M*_PHQ-9_ = 7.6, *SD*_PHQ-9_ = 7.0). There were no significant differences in age, gender, or PHQ-9 scores between the experimental and waitlist conditions (see Table 1).

### 2.2. Measures

#### 2.2.1. Patient Health Questionnaire 9-Item (PHQ-9)

The PHQ-9 is a questionnaire which measures the presence and severity of depression symptoms through the use of nine self-report questions that reflect the diagnostic criteria for Major Depressive Disorder [18, 19]. This measure was used in the present study to measure participants' experience of depressive symptoms at baseline and one week follow-up. An analysis of PHQ-9 scores was completed in a prior study [14].

*(i) Mood*. Participants' self-reported mood was measured using a nine-point Likert scale in the form of a slider ranging from “Extremely Negative” (1) to “Extremely Positive” (9).

*(ii) Enjoyment*. Participants' self-reported daily enjoyment was measured using a five-point Likert scale ranging from “Not at all” (1) to “Very much” (5).

**Figure 1:**
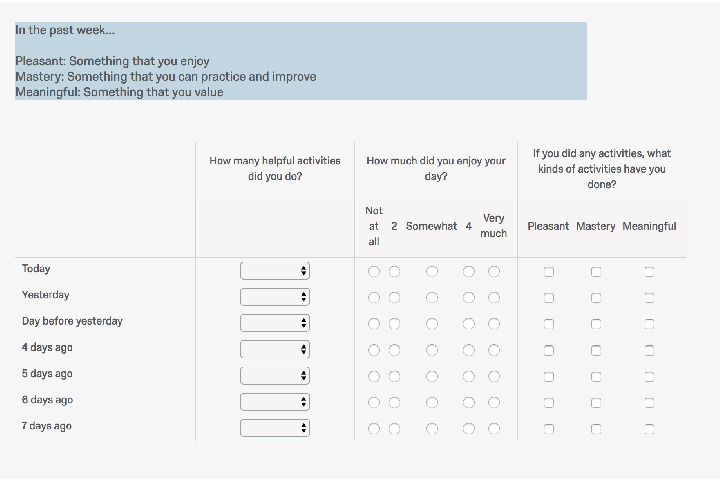
Type and frequency of activities per day.

*(iii) Activity Log*. Participants were asked to report on the past week at baseline and follow up with information about: number of activities done per day, enjoyment level, and the type of activities completed (see Figure 1). The number of activities done per day was completed with a drop-down menu. Types of activities were divided into three categories (Pleasant, Meaningful, and Mastery activities). Participants were provided with brief definitions of the different types of activities and participants were asked to indicate which type of activities they engaged in each day.

### 2.3. Procedures

At the outset of the study, participants were recruited via AMT and provided with a link to the site. Following consent, participants completed baseline questionnaire with demographic information including their age, gender, and country of origin. Additionally, participants were asked to complete an activity log divided into three categories: Pleasant, Meaningful, and Mastery; and the number of overall activities they engaged in over the course of the past week. Participants were asked to report their subjective level of enjoyment of that day and whether or not they engaged in activities. Pleasant activities were defined as “something you enjoy,” Mastery activities as “something you can practice and improve,” and Meaningful activities as “something that you value.”

Participants received a one week follow-up survey. This follow-up survey included questions asking participants to reflect on their motivation to change, confidence in being able to improve their mood. Additionally, participants were asked how carefully they completed the survey and the PHQ-9 compared to other studies. Results were compared to detect whether there was a significant change in these domains and experiences, and the directionality of that change.

### 2.4. Statistical Analysis

A temporal analysis model (fully described in [20]) was utilized to detect changes in daily enjoyment as a result of daily activity level. This analysis is a form of diary analysis that uses a mixed-effects autoregressive model to examine both a concurrent effect, as well as a lag effect of daily activity level on daily enjoyment. This was achieved by regressing daily enjoyment on the same day's activity level (concurrent), as well as the previous day's activity level (lag). In addition to these two effects, a concurrent∗lag interaction effect was also estimated, which models the effect of the previous day's activity level on the relationship between the concurrent day's activity level and concurrent day's enjoyment. Although the study involved an experimental and waitlist group, the current analysis is focused on the effect of different types of activities on mood and daily enjoyment. Additionally, there were no demographic differences between the groups. Therefore, the effect of condition was not included in the analyses.

A mixed-effects ANOVA was used to analyze the impact of time, number of activities, and age on participants' reported mood. The presence of depression (as measured by a continuous score on the PHQ-9) was controlled for in all analyses. Only participants in the experimental condition were included in this analysis because they received the activity log at both baseline and follow-up.

## 3. Results

Participants engaged in an average of 2.77 activities per day (Pleasant, Meaningful, and Mastery); males and females did not differ in this respect. Participants reported engaging in Pleasant activities on more days (*M* = 4.62 days per week), with Meaningful (*M* = 3.01), and Mastery (*M* = 2.98) activities being reported on fewer days of the week. Males and females differed only within Meaningful activities, with female participants reporting a greater number of Meaningful activities [*M*_diff_ = 0.69, *t *(314) = −2.28, *p* < .05] than male participants.

Regarding the total number of activities reported, higher PHQ-9 scores (*b* = −.21, *t *(241) = −4.58, *p* < .0001) predicted less engagement in helpful activities throughout the week. A main effect of age was also trending (*b* = −.04, *t *(240) = −1.76, *p* = .08), such that older participants were slightly less likely to engage in helpful activities.

### 3.1. Enjoyment

Due to the effect that depression levels and age had on the number of activities that participants engaged in, both variables were entered into the enjoyment analyses as covariates. The initial analysis regressed daily enjoyment on the number of helpful activities of the concurrent day as well as the number of helpful activities on the previous day. The concurrent∗lag interaction was also included. This analysis demonstrated a main effect of concurrent-day activities [*b* = 0.335, *t *(218) = 15.31, *p* < .001], such that a greater number of activities reported in a day predicted an increase in enjoyment on the same day. There was no significant lag effect of the previous day's activities, but the concurrent∗lag interaction was significant [*b* = –0.023, *t *(26.4) = –3.92, *p* < .001], suggesting that engagement in a higher number of activities on the previous day decreases the positive relationship between same-day activities and enjoyment. For example, engaging in seven activities today predicts an increase in daily enjoyment today. However, this may reduce the relative impact that five activities engaged in tomorrow will have on tomorrow's daily enjoyment.

The second analysis looked at the dichotomous (yes/no) answers to whether pleasant, mastery, or Meaningful activities were engaged in each day. The results suggested that all three types of activities significantly predicted concurrent enjoyment of that day (Pleasant: [*b* = 0.312, *t *(1811) = 46.73, *p* < .001]; Meaningful: [*b* = 0.254, *t *(1814) = 11.65, *p* < .001]; Mastery: [*b* = 0.290, *t *(1816) = 13.07, *p* < .001]). However, there was no lag effect or concurrent∗lag interaction, suggesting that while engaging in different types of activities affected enjoyment, this effect did not carry over to the next day.

### 3.2. Mood

**Figure 2:**
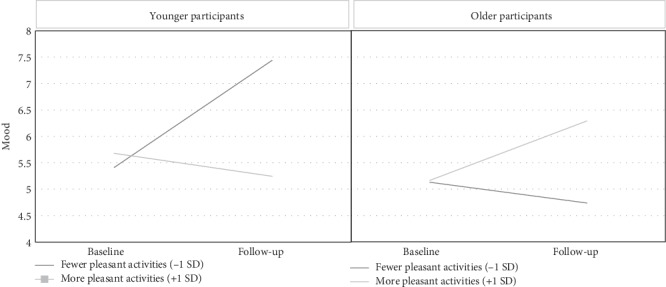
Three-way interaction between age, Pleasant activities, and time.

For the mood analysis, mood level was regressed on time as well as the number of days each activity was reportedly engaged in throughout the week. Age and depression level were also entered into the analysis as covariates. Only Mastery activities predicted mood [F (1,39) = 4.89, *p* < .05], such that the more days a participant reported engaging in Mastery activities, the higher their reported mood was at baseline and follow-up. There was, however, a significant three-way interaction between age, Pleasant activities, and time [F (1,26) = 4.42, *p* < .05]. For younger adults (1 SD below the age mean) who engaged in Pleasant activities fewer days per week (1 SD below the activities-per-day mean), there was an improvement in mood from baseline to follow-up. However, younger adults (1 SD above the age mean) who engaged in Pleasant activities more days per week (1 SD above the activities-per-day mean) experienced a small deterioration in mood from baseline to follow-up. Older adults (1 SD above the age mean) exhibited an opposite, but more predictable, pattern. While older adults exhibited similar mood levels at baseline regardless of activity level, those who engaged in Pleasant activities on more days per week reported a significantly better mood at follow-up than in older adults who engaged in Pleasant activities fewer days per week (see Figure 2).

## 4. Discussion

Behavioral Activation (BA) is a well-established intervention for depression [5, 7]. BA assumes that engaging in Pleasant activities is a first step in facilitating a patient's shift from a depressive state to a healthy mood state, and Mastery activities can contribute to improve individuals' mood and produce changes in their environment. Additionally, previous studies have shown that engaging in Meaningful activities is a key component in successfully treating depression [9].

Findings showed that participants reported engaging in Pleasant activities on more days, followed by Meaningful, and then Mastery activities. Regarding the impact of activities on same-day enjoyment, the three types of activities (Pleasant, Meaningful, and Mastery) demonstrated a significant effect. This indicates that engaging in any of these helpful activities positively impacted the participant's enjoyment of that day. A greater number of overall activities (regardless of the type of activity) reported on a given day led to an increase in enjoyment that day and the following day. However, this increase in enjoyment was not found to be as impactful on the following day. This may be suggestive of the presence of a satiation effect, wherein an individual may need to increase their daily dose to achieve the same impact on the following day's enjoyment. Overall, there appears to be an immediate effect of activities on enjoyment but not an enduring effect. The diminished impact of activities on the following day highlights the importance of finding a stable number of activities per day to achieve a daily sustained enjoyment level.

Regarding the impact of all three types of activities in participant's mood, only Mastery activities predicted mood; the more days a participant reported engaging in Mastery activities, the higher their reported mood was at follow-up. This suggests that engaging in Mastery activities is the most impactful on mood and therefore should be highlighted in BA interventions for individuals experiencing depression. This finding combined with the result that the higher a participant's depression level, the less likely they are to engage in Pleasant and Meaningful activities, may suggest that augmenting Mastery activities for those with depression may be a useful approach.

When the role of age, mood, and type of activities was assessed, a significant three-way interaction between age, Pleasant activities, and time was observed. The three-way interaction revealed that the younger adults, who engaged in fewer Pleasant activities per week experienced an improvement in their mood, compared to the observed worsening in mood in younger adults that engaged in more Pleasant activities per week. Interestingly, older adults showed the predicted pattern, that is, more activities were associated with a better mood. These two patterns, suggest that clinical recommendations based on Pleasant activities could vary by age, and that efforts towards the increase of Pleasant activities may be more relevant to older adults.

Finally, when gender differences were explored, female participants reported engaging in Meaningful activities on a greater number of days than male participants. The study design does not allow the determination of whether female participants actually engaged in more Meaningful activities or if they perceived activities as being more Meaningful.

### 4.1. Limitations and Future Directions

There were logistical limitations regarding the nature and frequency of measuring certain variables in the survey. For example, mood was assessed at two time points (baseline and follow-up), and enjoyment was reported retrospectively for every day at follow-up. The type and frequency of activities per day assessed the total number of all types of activities an individual participated in every day, but do not specify the quantity of each type. Additionally, the study was retrospective in nature and relied purely on participants' ability to recall their mood, daily activities, and daily enjoyment.

In future research, it may be beneficial to gather information on the number of activities an individual engaged in within each type of activity (i.e., Pleasant, Meaningful, and Mastery), even including a qualitative component of what specific activities each person engaged in. This with more detailed assessment of the quantity and ratio of types of activities engaged in per day may enable a more fine-tuned examination of the impact on mood and daily enjoyment. Future studies may also benefit from gathering participant mood and enjoyment ratings daily, rather than retrospectively, and at the same time-points to increase the accuracy of ratings.

## 5. Conclusions

Overall, this study supports the therapeutic belief that engagement in activities can have a positive impact on mood and that specifically Mastery activities may be particularly beneficial in mood improvement. Interestingly the effect of Pleasant activities had a positive effect on time for older adults but not for younger adults, and female participants reported engaging in more Meaningful activities. These findings contribute to the understanding on how BA may influence participant's mood and enjoyment, and can inform clinician's recommendations about the types of activities. Overall, this study suggests that not all activities are created equal and that Mastery activities may be more capable of contributing to mood improvement.

## Figures and Tables

**Table 1:** Participant demographics.

Characteristic	All	Experimental	Waitlist
	(*n* = 684)	(*n* = 338)	(*n* = 346)
*Gender*	*n* = 683	*n* = 338	*n* = 345
Male	222 (32.5%)	106 (31.4%)	116 (33.6%)
Female	461 (67.5%)	232 (68.6%)	229 (66.4%)
*PHQ-9 severity*	*n* = 580	*n* = 285	*n* = 295
None	217 (37.4%)	116 (40.7%)	101 (34.2%)
Mild	137 (23.6%)	68 (23.9%)	69 (23.4%)
Moderate	111 (19.1%)	55 (19.3%)	56 (19.0%)
Moderately severe	72 (12.4%)	31 (10.8%)	41 (13.9%)
Severe	43 (7.4%)	15 (5.3%)	28 (9.5%)
*Age*	*n* = 684	*n* = 338	*n* = 346
M (SD)	35.8 (12.6)	36.4 (12.5)	35.3 (12.3)

Note: No differences were significant.

## Data Availability

The data used to support the findings of this study are available from the corresponding author upon request.
